# Long Term Expansion of Bone Marrow-Derived hMSCs on Novel Synthetic Microcarriers in Xeno-Free, Defined Conditions

**DOI:** 10.1371/journal.pone.0092120

**Published:** 2014-03-17

**Authors:** Martial Hervy, Jennifer L. Weber, Marylene Pecheul, Paula Dolley-Sonneville, David Henry, Yue Zhou, Zara Melkoumian

**Affiliations:** 1 Corning Life Sciences Development, Corning Incorporated, Corning, New York, United States of America; 2 Corning European Technology Center, Corning Incorporated, Avon, France; Indian Institute of Toxicology Research, India

## Abstract

Human mesenchymal stem cells (hMSCs) present an attractive target for cell therapy given their wide availability, immunomodulatory properties, and multipotent nature for differentiation into chondrocytes, osteocytes, and adipocytes. With the progression of hMSC clinical studies, there is an increasing demand for development of technologies that enable efficient cell scale-up into clinically relevant quantities. Commercial scale manufacturing of hMSCs will require a large surface area which is not cost effective with available two-dimensional culture vessels. Recent studies showed that microcarriers provide a three-dimensional culture environment suitable for hMSC expansion. Traditionally, biological coatings and/or serum-containing medium are required to facilitate hMSC attachment and expansion in dynamic conditions. These limitations may hinder the use of microcarriers as a scale-up technology for hMSC therapeutics, where cell products, and therefore patient safety, are more controlled with the use of xeno-free, defined culture conditions. Here we report the long term culture of hMSCs on novel synthetic Synthemax II microcarriers in two different xeno-free media. Cells were maintained over 40 days on sterile, ready-to-use microcarriers in spinner flasks with programmed agitation. hMSC expansion was obtained by addition of fresh beads without the need for enzymatic dissociation. We achieved a cumulative cell expansion of >10,000 fold, and cells retained normal hMSC phenotype, karyotype, and tri-lineage differentiation potential. To our knowledge, this report is the first example of long term culture of hMSCs on synthetic microcarriers in xeno-free, defined conditions.

## Introduction

Human mesenchymal stem cells (hMSCs) are multipotent adult stem cells able to differentiate to adipogenic, osteogenic or chondrogenic lineages [1]; the properties of hMSCs make them attractive cell therapy agents. Pre-clinical studies identified hMSCs for the treatment of various pathologies including acute lung injury [2], septic shock [3], and myocardial infarction [4]. In addition, ongoing clinical trials are investigating hMSC therapy in graft versus host diseases [5], cardiac pathologies [6], and cancers [7]. These studies suggest that hMSC therapeutic efficacy is a result of immunomodulatory and paracrine events, and the use of hMSCs as therapeutic agents presents minimal risk for adverse side effects [8].

hMSCs can be isolated from various sources including bone marrow, adipose tissue and placenta; however, the quantity of cells purified is small compared to the large therapeutic dosage required for autologous therapies (up to 2×10∧8 cells per kg per dose [9]). Similarly, cell quantities would increase exponentially in large-scale production for allogeneic cell therapies. Therefore, extensive expansion of hMSCs is required to obtain therapeutic cell numbers from purified cells. Given that hMSCs are adherent and contact inhibited, current methods for cell scale-up involve single or multi-layer vessels, which require labor intensive manipulations and present difficultly when monitoring pH, nutrient consumption, and gas exchange.

An alternative approach utilizes microcarrier-based stirred cultures in spinner flasks or bioreactor systems. Microcarriers are generally small, spherical particles (100-400 μm) that function as the adhesion substrate for cells cultured in a stirred environment. Due to the three-dimensionality of microcarriers, they offer a large surface area for cell expansion in a limited footprint (up to 15000 cm^2^/liter of culture for vaccine applications [10]). Commercially-available microcarriers include two common classes: rigid particles made of glass or plastic (polystyrene) and soft, swellable particles (gelatin, alginate, or dextran). Both types can be functionalized chemically or coated with extracellular matrix (ECM) proteins to further promote cell adhesion.

Recent studies demonstrated hMSC expansion using microcarrier-based culture systems. Collagen-coated Cytodex-3 (GE Healthcare) and gelatin-coated CultispherS (Percell) microcarriers are most commonly used with success for hMSC production in serum-containing media [11], [12] [13]. Although these studies define optimized culture conditions for large-scale hMSC production, they require biological coatings and serum-containing medium to facilitate cell attachment and expansion in stirred conditions. In addition, the microcarriers require time-consuming and labor intensive preparation (e.g. pre-swelling in water or buffer, autoclave sanitization) prior to cell seeding. These limitations hinder the use of microcarriers for hMSC therapeutics, where cell products are more reproducible with the use of defined culture conditions [14] [15].

Here we report the long-term expansion of hMSCs on synthetic microcarriers in defined, xeno-free media. Cells were maintained for multiple passages on sterile, ready-to-use Corning Synthemax II-coated microcarriers in spinner flasks. Cells retained typical, spindle-like morphology, phenotypic marker expression, and *in vitro* tri-lineage differentiation potential on Synthemax II microcarriers in two different defined, xeno-free media. Cell expansion was obtained through direct addition of fresh microcarriers; cells migrated from bead-to-bead without the need for cell re-seeding or enzymatic dissociation. We achieved successful expansion (>10,000 cumulative fold expansion in 40 days) of hMSCs on synthetic microcarriers in defined, xeno-free media. Further, we demonstrate that cells present normal karyotype after long term culture on Synthemax II microcarriers.

## Materials and Methods

### Cells

Human bone marrow-derived mesenchymal stem cells were obtained from STEMCELL Technologies (Cat. No. MSC-001F). For routine maintenance in Mesencult-XF medium (STEMCELL Technologies, Cat. No. 05429), cells were grown on Mesencult-XF attachment substrate (STEMCELL Technologies, Cat. No. 05424) coated plates. For routine maintenance in Corning stemgro hMSC medium (Mediatech Inc., Cat. No. 40-410-CV), cells were grown on Corning CellBIND T75 flasks.

### Microcarrier preparation

Sterile, ready-to-use Corning Synthemax II microcarriers (Corning, Cat. No. 3781) were reconstituted in water according to manufacturer’s instructions and used directly. Water was removed, and microcarriers were diluted with culture medium prior to cell seeding.

### Spinner flasks cultures

Synthemax II microcarriers (500 mg) were resuspended in 10 mL culture medium to achieve a density of 15 cm^2^/mL. Microcarriers were transferred to Corning 125 mL disposable spinner flasks (Corning, Cat. No. 3152). hMSCs were seeded directly onto Synthemax II microcarriers at 5000–6000 cells per cm^2^ in a 15 mL final volume (0.75-1E6 cells per spinner flask). Spinner flasks were placed in a cell culture incubator at 37°C under a 5% CO_2_ atmosphere, without agitation for 18–20 hours. After that, medium volume was adjusted to 35 mL (up to 45 mL performed similarly), and cultures were stirred at 30 rpm every 2 hours for 15 minutes. Culture medium (50–60%) was replaced every two days.

1. **One passage study with growth kinetic.** hMSCs were seeded onto microcarriers as described previously. Daily, cell-microcarrier samples were taken from each spinner flask culture. Samples were washed twice with dPBS prior to treatment with trypsin-EDTA for 5 minutes. Microcarriers were separated from cells either by decantation or cell strainer (BD Biosciences, Cat. No. 352350). Cells were stained with trypan blue and counted using a hemacytometer or an automated cell number/viability analyzer, Vi-Cell (Beckman Coulter).
2. **Serial passage in Mesencult XF Medium.** hMSC seeding was performed as described above. After 7 days, cells were passaged by removing 80% of the culture after gentle homogenization. Fresh Synthemax II microcarriers (500 mg) were added back to the spinner flask. The final culture volume was adjusted to 35 mL with Mesencult XF medium, and agitation was started at 30 rpm for 15 minutes every 2 hours. Collected cells-microcarriers were pelleted by centrifugation, washed with dPBS, and treated with trypsin. Detached cells were counted using the trypan blue exclusion method with a hemacytometer. Duplicate cell samples were collected for flow cytometry analysis and directed differentiation experiments.
3. **Thaw and serial passaging in stemgro hMSC Medium.** hMSCs from a working cell bank were removed from liquid nitrogen storage and thawed in 37°C water bath. Cells were pelleted to remove dimethyl sulfoxide (DMSO) containing freezing medium, reconstituted in pre-warmed stemgro hMSC Medium, and seeded directly onto microcarriers at 5000 cells per cm^2^ as described above. After 4–5 days, cells were passaged by removing 90–95% of the culture to achieve the density of 3500 cells per cm^2^. Fresh Synthemax II microcarriers (500 mg) were added back to the spinner flask. The final culture volume was adjusted to 45 mL with stemgro hMSC Medium, and agitation was started at 30 rpm for 15 minutes every 2 hours. Collected cells were removed from microcarriers with trypsin-EDTA and then strained through 70 micron filter to remove microcarriers prior to cell count with the Vi-Cell. Duplicate cell samples were collected for flow cytometry analysis and directed differentiation experiments.

### Flow cytometry

Harvested cells were fixed in 2% paraformaldehyde in dPBS for 10 minutes at room temperature and then washed and stored in dPBS. Cells were incubated in blocking buffer for 15 min at 4°C, followed by 30 minute incubation at 4°C with primary antibodies against CD14, CD45, CD73, CD105 (Millipore Chemicon) or corresponding isotype controls (Invitrogen/Molecular Probes) in blocking buffer. After a brief wash, cells were incubated with 1∶800 dilution of corresponding secondary antibodies for 30 minutes at 4°C in the dark. For each sample, 30,000 events were acquired using BD FACS Calibur Flow Cytometer (BD Biosciences). Histogram overlay subtraction analysis using the BD Cell Quest Pro software (BD Biosciences) was performed to calculate the percent of marker positive cells.

### Directed differentiation

Cells were harvested from microcarriers after 7 passages in defined, xeno-free medium and re-plated on 6-well plates for directed differentiation into adipogenic, osteogenic, and chondrogenic lineages using the following kits: Adipogenic differentiation kit (Lonza, Cat. PT3102A/B), StemPro Osteogenic differentiation kit (Gibco, Cat. No. A10072-01) and StemPro Chondrogenic differentiation kit (Gibco, Cat. No. A10071-01). Stains used: Oil Red Staining Kit (Millipore), Alizarin Red Stain (Millipore), and 1% Alcian Blue Stain (Fisher Scientific). The protocols for differentiation and staining were performed following manufacturer’s recommendations.

### Karyotype analysis

At passage 7, cells were harvested from microcarriers using trypsin/EDTA and re-seeded onto Synthemax T75 flasks at 3500 cells per cm^2^. Day 2 live cultures were sent to WiCell Cytogenetics Laboratory for karyotype analysis.

## Results

### Synthetic Synthemax II microcarriers support hMSC attachment and short term expansion in two defined, xeno-free media

Previous studies [11] [12] [13] have shown that hMSCs can be maintained on microcarriers in the presence of serum-containing medium or biological coatings. Here, we propose to eliminate these animal-derived components in the culture of hMSCs on microcarriers. To evaluate novel synthetic microcarriers in defined, xeno-free media, we examined cell attachment and expansion in dynamic culture conditions over one passage.

**Figure 1:**
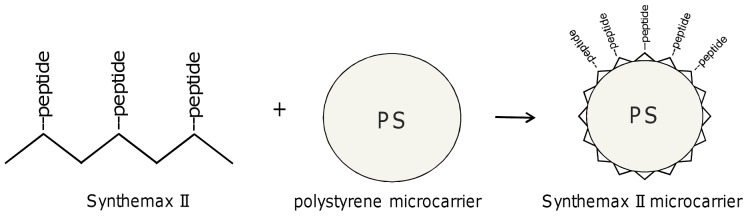
Schematic representation of Synthemax II microcarrier preparation, by incubation of polystyrene microcarriers with peptide grafted Synthemax II polymer.

**Figure 2:**
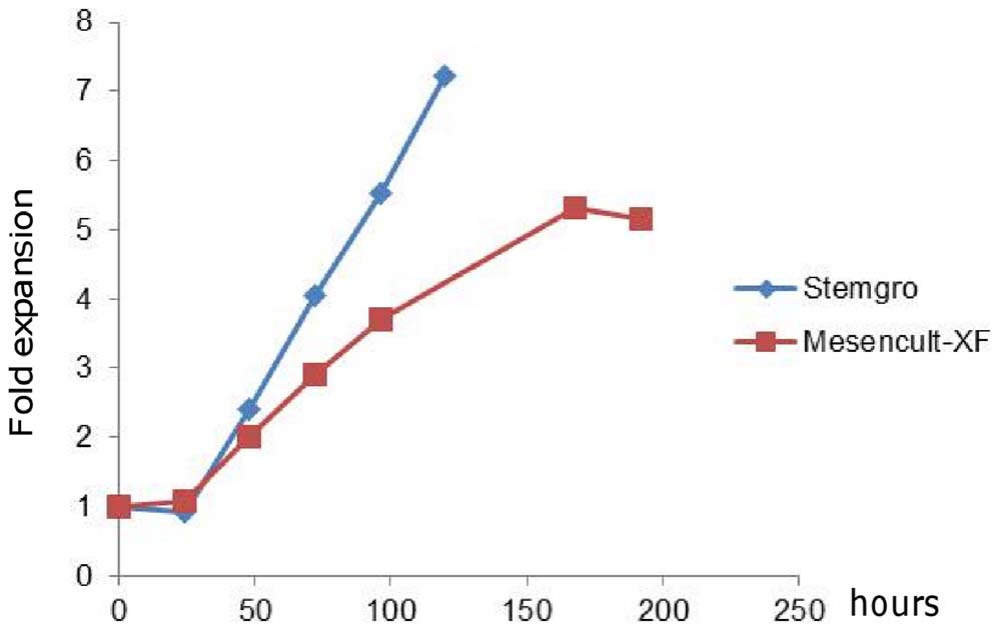
Growth curve for hMSCs on Synthemax II microcarriers in spinner flasks in Mesencult XF and hMSC stemgro media. hMSCs were seeded directly onto Synthemax II microcarriers at 5000–6000 cells per cm^2^ in a 15 mL final volume in 125 mL spinner flasks. Cultures were placed in an incubator at 37°C under a 5% CO_2_ atmosphere, without agitation for 18–20 hours. After that, medium volume was adjusted to 35 mL, and cultures were stirred at 30 rpm every 2 hours for 15 minutes. Culture medium (50–60%) was replaced every two days.

Synthemax II microcarriers (Corning) are polystyrene microcarriers modified with a synthetic polymer and adhesion peptide, as shown in Figure 1. Microcarriers are supplied sterile and ready-to-use from the manufacturer. hMSCs were seeded on 500 mg Synthemax II microcarriers in spinner flasks (150 cm^2^ per flask), as described in Methods. Duplicate cultures were maintained in two commercially available, xeno-free media: Mesencult XF (STEMCELL Technologies) medium and stemgro hMSC (Corning) medium. Cell adhesion occurred in static condition for the first 20 hours, after which cultures were exposed to intermittent agitation at 30 rpm for 15 min every 2 hours. A sample of each culture was collected for cell enumeration every day for 5–7 days, depending on confluence. As shown in Figure 2, cell growth lagged for 24 hours, which corresponds to initial cell attachment and adaptation to culture conditions. We observed 80–90% of cells attached to the microcarriers after 24 hours in both Mesencult XF and stemgro hMSC media, and exponential growth was seen from days 2–5. After 5 days, Synthemax II microcarriers supported 5 fold cell expansion in Mesencult XF medium and 7 fold expansion in stemgro medium (Figure 2), confirming hMSC growth on Synthemax II microcarriers in xeno-free, dynamic conditions. Further, cell expansion on Synthemax II microcarriers in stemgro hMSC medium was compared for hMSCs from 3 different donors; after 4 days in dynamic culture, 6–9 fold cell expansion was achieved (data not shown).

### Synthemax II microcarriers support the long term expansion of hMSCs in defined, xeno-free media

**Figure 3:**
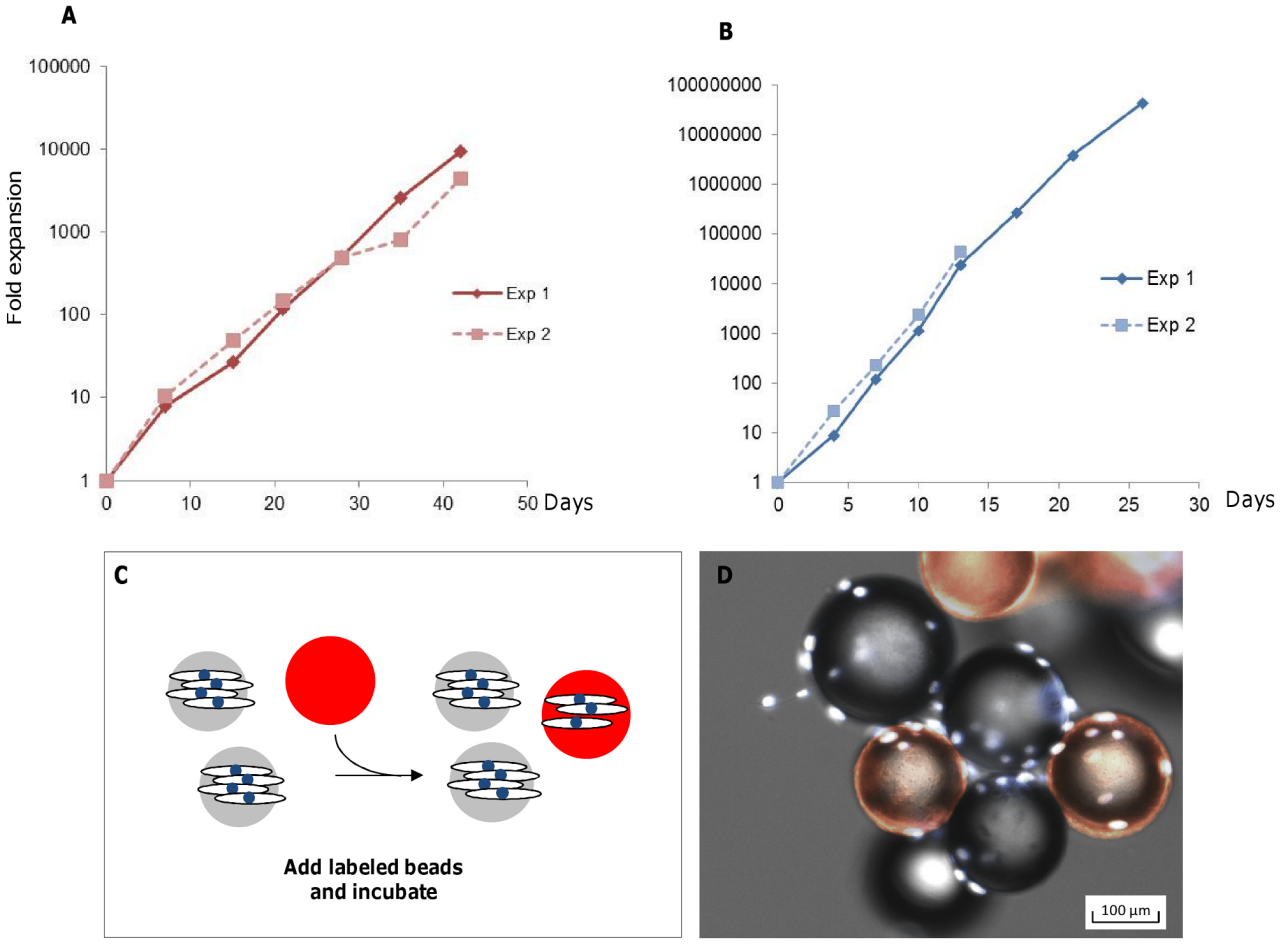
hMSC long term expansion on Synthemax II microcarriers in Mesencult XF and hMSC stemgro media. Growth curves show the net projected cell expansion for hMSCs on Synthemax II microcarriers in Mesencult XF medium (A) and hMSC stemgro medium (B). Stemgro replicate experiment (Exp 2) was terminated after 4 passages, with healthy cells. (C) A schematic representation of cell migration from bead to bead, where cells migrate to freshly added, rhodamine-labeled microcarriers (shown in red). (D) Fluorescent microscopy image illustrating cell migration to freshly added microcarriers. Cell (nuclei stained with DAPI (white)) have migrated from existing microcarriers (grey) to freshly added rhodamine-labeled microcarriers (red).

Next, we examined the ability of Synthemax II microcarriers to support expansion of hMSCs over several cell passages. Using the methods described above, hMSCs were seeded on microcarriers in spinner flasks until 80% confluence (4–7 days depending on medium). Cell viability and expansion rate were assessed at the end of each passage by collecting cell-microcarrier samples from each spinner flask. Cells were released from microcarriers using trypsin-EDTA, and microcarriers were separated from cells via decantation or a cell strainer. Based upon the total cell number per spinner flask, ∼80–90% of the culture was removed, and an equal amount of fresh microcarriers was added to maintain a surface area of 150 cm^2^ per spinner flask. This method of bead-to-bead cell expansion was used to maintain hMSC cultures for 7 consecutive passages on Synthemax II microcarriers in Mesencult XF and stemgro media. Figure 3 shows the net fold expansion in replicate experiments, after 42 days in Mesencult XF (Figure 3A) and 26 days in stemgro hMSC medium (Figure 3B). We observed an average of 2.3±0.4 and 3.6±0.4 population doublings per passage for cells grown in Mesencult XF and stemgro hMSC medium, respectively. Greater than 90% of the cells were viable for both media conditions (data not shown). Note that a higher rate of cell expansion was observed for cells maintained in stemgro hMSC medium compared to those in Mesencult XF, which may be attributed to the difference in medium composition or the fact that the experiments were not performed side-by-side or in the same facility.

### Bead-to-bead transfer is an efficient method for hMSC expansion in defined, xeno-free media

Traditionally on two-dimensional surfaces, cells are subcultured using enzymes that digest protein interactions between cells and the surface. However, the repeated use of enzymes during cell passaging can damage cellular receptors required for cell attachment and intracellular communication. We investigated a method for cell expansion by addition of new microcarriers to promote cell migration and growth in defined, xeno-free media without the need for enzyme dissociation. To verify that cells migrate to fresh, uninhabited microcarriers, we coated the fresh microcarriers with rhodamine-labeled Synthemax II. Cells were seeded onto non-labeled Synthemax II microcarriers in a spinner flask, as described previously. Rhodamine-labeled Synthemax II microcarriers were added to the culture as illustrated in Figure 3C. After 24 hours of intermittent agitation, a sample was transferred to a 24-well plate. Cells were stained with nuclear dye, DAPI, and imaged on microcarriers using fluorescence microscopy. As shown in Figure 3D, cells have colonized the rhodamine-labeled microcarriers (red), indicating that cells migrate to fresh microcarriers with intermittent agitation.

### hMSCs retain phenotypic marker expression after long term expansion on Synthemax II microcarriers in defined, xeno-free media

**Figure 4:**
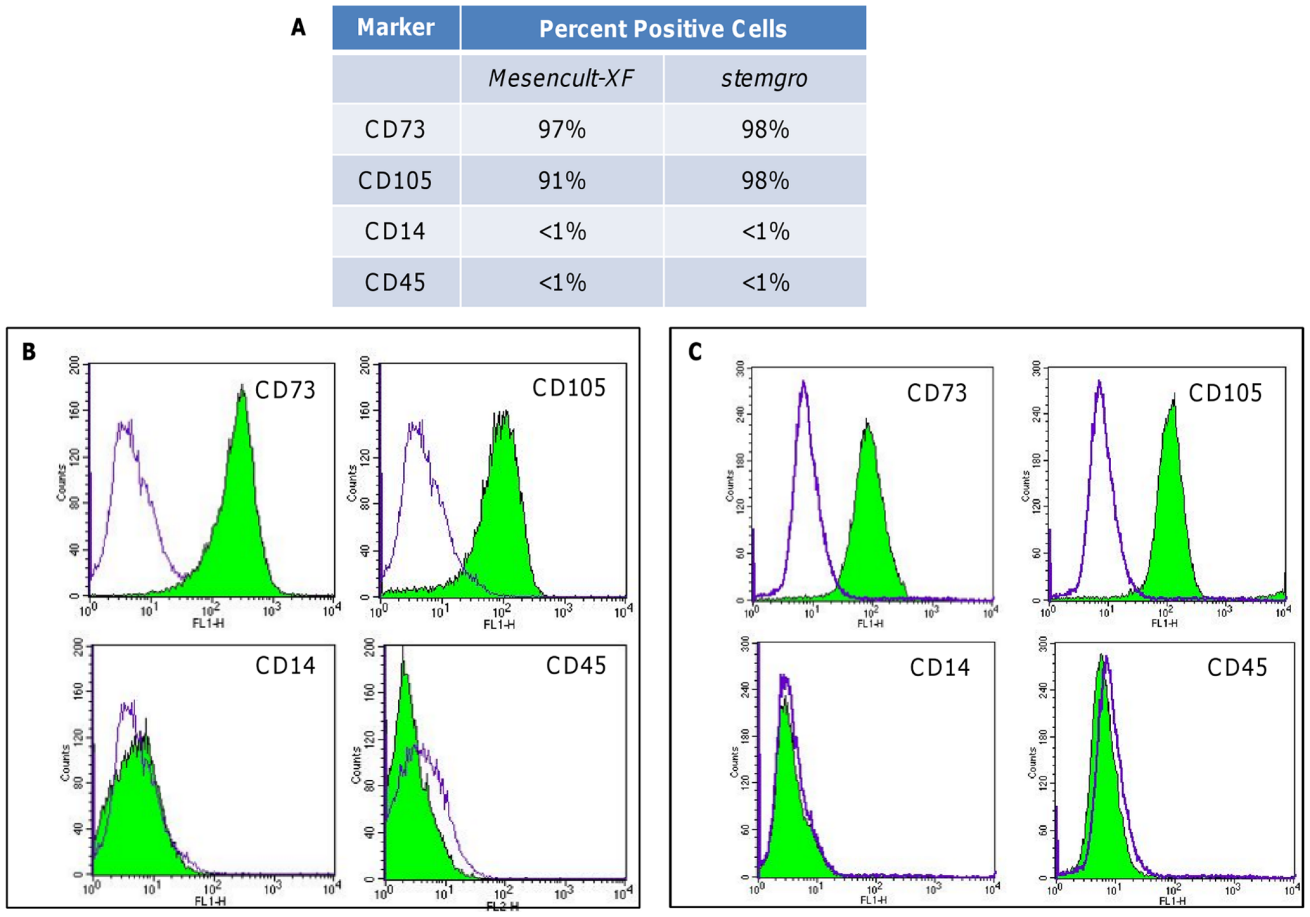
hMSCs retain typical phenotypic marker expression after 5 passages on Synthemax II microcarriers in xeno-free media. (A) Quantitative marker analysis showing percent of cells expressing CD73, CD105, CD14 and CD45 after long term expansion Synthemax II microcarriers in Mesencult XF and hMSC stemgro media. Flow cytometry histograms for each phenotypic marker are shown for cells maintained on Synthemax II microcarriers in Mesencult XF medium (B) and hMSC stemgro medium (C).

hMSC phenotype was assessed after 5 passages on Synthemax II microcarriers in xeno-free media. Briefly, cells were removed from microcarriers using trypsin-EDTA, and microcarriers were separated via decantation or a cell strainer. Cells were fixed in paraformaldehyde and stained for specific phenotypic markers prior to flow cytometry analysis. Quantitative phenotypic marker expression for hMSCs maintained on Synthemax II microcarriers in Mesencult XF and stemgro hMSC media is shown in Figure 4A; histogram analysis for each marker is shown in Figure 4B (Mesencult XF medium) and 4C (stemgro hMSC medium). CD105 and CD73 markers were expressed in >90% of the cells and CD14 and CD45 markers were expressed in <1% of the cells for both media conditions, confirming typical hMSC phenotype after long-term culture on Synthemax II microcarriers. Importantly, hMSC surface marker expression was comparable for cells cultured on Synthemax II microcarriers and two-dimensional cultures in flasks (data not shown).

### hMSCs maintain spindle-like morphology and normal karyotype after expansion on Synthemax II microcarriers in xeno-free media

**Figure 5:**
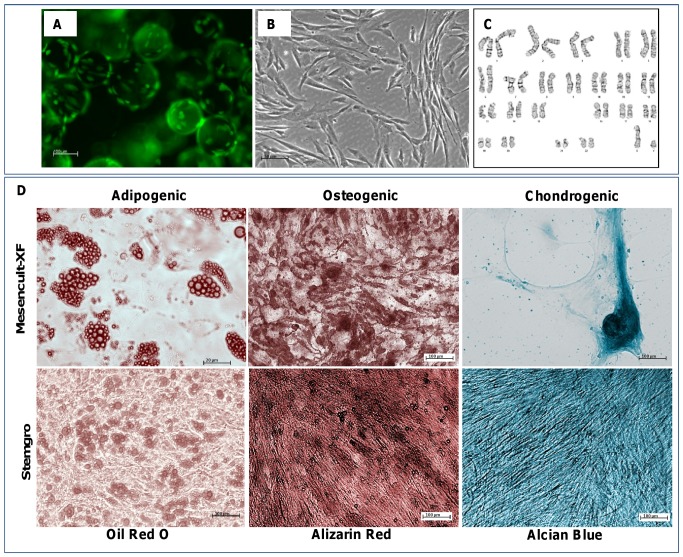
Cell characterization after long term growth on Synthemax II microcarriers in xeno-free medium. (A) Fluorescent image of hMSCs on Synthemax II microcarriers after calcein AM staining. (B) Phase image of hMSCs after migration off Synthemax II microcarriers to a 6-well plate. (C) hMSC karyotype analysis after 7 passages on Synthemax II microcarriers in stemgro hMSC medium. (D) After 5 passages on Synthemax II microcarriers, hMSCs were induced to differentiate into adipogenic, osteogenic, and chondrogenic lineages and were stained with oil red O, alizarin red, and alcian blue, respectively. Representative images of stained cells from both media are shown.

To confirm that hMSC morphology is maintained during and after long term passage on Synthemax II microcarriers in defined, xeno-free media, cells on microcarriers were stained with a viability marker, calcein AM. As shown in Figure 5A, cells retain their elongated morphology, and we observed uniform cell confluence and distribution across all microcarriers. To better visualize cell morphology under bright field microscopy, after 7 passages on Synthemax II microcarriers in stemgro hMSC medium, cells on microcarriers were added to a 6-well plate for static culture. As shown in Figure 5B, cells migrated off of the microcarriers and expanded to ∼80% confluence by day 3, while retaining an elongated morphology. In addition, normal karyotype was confirmed for hMSCs maintained for 7 passages on Synthemax II microcarriers in stemgro hMSC medium (Figure 5C).

### hMSCs retain tri-lineage differentiation potential after expansion on synthetic microcarriers in defined, xeno-free media

To confirm that hMSCs maintain their multipotency after long term expansion on Synthemax II microcarriers, cells were removed from microcarriers and re-seeded in 6 wells plates. Cells were induced to differentiate into adipogenic, osteogenic, and chondrogenic lineages using commercially available cell differentiation kits. After three weeks, cells were fixed in paraformaldehyde and stained for markers of each lineage type. Adipogenic, osteogenic, and chondrogenic lineages were stained with oil red O, alizarin red, and alcian blue, respectively. Representative images of stained cells are shown in Figure 5D. Effective cell differentiation into each lineage was observed for cells maintained on microcarriers in Mesencult XF and stemgro hMSC media, confirming that cells grown on Synthemax II microcarriers in defined, xeno-free media retain their multipotency.

## Discussion

In this study we report a method for large scale expansion of human mesenchymal stem cells on synthetic microcarriers in defined, xeno-free culture medium. We demonstrate hMSC multi-passage expansion and multipotency on Synthemax II microcarriers in two different defined, xeno-free media. Further, cell scale-up was accomplished via bead-to-bead expansion by addition of fresh microcarriers without the need for enzymatic dissociation.

To enable consistent and robust expansion of hMSCs, cultures must contain adequate sources of cell adhesion and growth promoting factors. To this end, recent studies have focused on the use of serum supplementation and/or extracellular matrix (ECM) protein-coatings. Collagen-coated Cytodex 3 [11] and gelatin-coated Cultispher S [12] [13] microcarriers have been used in combination with serum-containing medium to achieve high cell expansion rates. These studies demonstrated potential culture conditions for expansion of hMSCs on microcarriers, but cell attachment and growth remained dependent upon animal-derived coating and media supplements. The use of serum has several limitations including: batch to batch variability and availability, possible contamination with pathogens, and the presence of known and unknown components which affect cell morphology, growth rate, function, and phenotype [16]. Dos Santos et. al. reported the use of gelatin Cultispher S or ECM-coated microcarriers for hMSC growth on microcarriers in serum-free medium; in this study, cells required a biological coating for adequate cell adhesion and expansion [17]. Ideal culture conditions for certain applications require a serum-free, defined medium that supports cell attachment and expansion while maintaining intrinsic cell qualities.

In this study, we evaluate xeno-free culture conditions for the expansion of hMSCs on microcarriers. Additional experiments are required to compare cell expansion in this environment to that in serum-containing medium and/or on biological-coated microcarriers. Future studies should aim to optimize the xeno-free culture conditions to maximize cell expansion while retaining the innate properties of hMSCs. Here, we report the expansion of hMSCs on novel synthetic microcarriers in two defined, xeno-free media: Mesencult XF and stemgro hMSC medium. Cell attachment to microcarriers was enabled through the vitronectin peptide-functionalized surface, Synthemax II, which is similar to the Synthemax synthetic surface used for both the hESC culture in xeno-free medium described by Melkoumian et al. [18] and the hMSC culture described by Dolley-Sonneville et al. [19]. Synthemax II microcarriers are synthetic, xeno-free, gamma sterilized, and require no preparation prior to use, in contrast to many other commercially available microcarriers that require pre-swelling and sterilization by autoclaving. The ready-to-use properties of Synthemax II microcarriers can significantly reduce the labor time and the risk of contamination associated with additional manipulation of microcarriers prior to cell seeding. The combination of Synthemax II microcarriers and serum-free medium provides a defined, xeno-free culture condition for hMSC expansion.

In addition, we observed spontaneous bead-to-bead expansion of hMSCs on Synthemax II microcarriers. Bead-to-bead transfer enables the efficient scale-up of hMSCs without the need for traditional enzymatic cell dissociation from microcarriers. hMSC migratory behavior in 2-dimensional vessels has been well documented [20], and we observed similar cell migration from microcarrier to microcarrier (Figure 3D) with sufficient contact time. Cell expansion improves with a static period(s) during culture [21] [22] to allow cells to colonize fresh microcarriers [23]. To this end, we implemented an intermittent agitation protocol. Studies have shown that intermittent agitation is sufficient for homogeneous nutrient and gas distribution, reduces shear stress, which can be problematic for hMSCs [24] [25] [26], and prevents excessive formation of cell-bead aggregates. Limited aggregation is reported by most studies, independent of agitation protocol, and recent publications by dos Santos et al. [17] and Ferrari et al. [27] suggest that addition of fresh microcarriers to the culture may be a more efficient method to control aggregate formation. We observed cell-microcarrier aggregates as the cell density of the culture increased, and as seen in Ferrari’s work, the aggregate size decreased after addition of fresh microcarriers. The formation of small cell-microcarrier aggregates is crucial for bead-to-bead migration and should not be completely eliminated or prevented. Furthermore, when we compared intermittent and continuous agitation protocols using the same agitation speed, we did not observe diminution in aggregate size or an increase in growth rate with continuous agitation (data not shown).

In summary, our results demonstrate successful expansion of hMSCs on Synthemax II microcarriers in defined, xeno-free media via bead-to-bead transfer without the need for enzyme dissociation. Cells retained high viability, characteristic morphology, phenotype, and tri-lineage differentiation capability when maintained for multiple passages on Synthemax II microcarriers. To our knowledge, this report is the first example of long term culture of hMSCs on synthetic microcarriers in xeno-free, defined conditions.
